# Caucasian Dragonheads: Phenolic Compounds, Polysaccharides, and Bioactivity of *Dracocephalum austriacum* and *Dracocephalum botryoides*

**DOI:** 10.3390/plants11162126

**Published:** 2022-08-15

**Authors:** Nina I. Kashchenko, Gunay S. Jafarova, Javanshir I. Isaev, Daniil N. Olennikov, Nadezhda K. Chirikova

**Affiliations:** 1 Laboratory of Medical and Biological Research, Institute of General and Experimental Biology, Siberian Division, Russian Academy of Science, 670047 Ulan-Ude, Russia; 2 Department of Pharmacognosy, Azerbaijan Medical University, Anvar Gasimzade Street 14, AZ1022 Baku, Azerbaijan; 3 Department of Biochemistry and Biotechnology, North-Eastern Federal University, 58 Belinsky Street, 677027 Yakutsk, Russia

**Keywords:** *Dracocephalum austriacum*, *Dracocephalum botryoides*, liquid chromatography-mass spectrometry, metabolomics, flavonoids, polysaccharides, antioxidants

## Abstract

*Dracocephalum botryoides* Steven and *Dracocephalum austriacum* L. are unexplored species of the *Dracocephalum* genus (Lamiaceae family) with a distribution in the Caucasus, where they are used in folk medicine and local cuisine. There are no data on the chemical composition of these *Dracocephalum* species. In this study, the application of a liquid chromatography-mass spectrometry technique for the metabolite profiling of methanol extracts from herbs and roots of *D. austriacum* and *D. botryoides* resulted in the identification of 50 compounds, including benzoic acid derivatives, phenylpropanoids, flavonoids and lignans. Water-soluble polysaccharides of the herbs and roots of *D. austriacum* and *D. botryoides* were isolated and characterized as mostly pectins with additive arabinogalactan-protein complexes and starch-like compounds. The antioxidant potential of the studied extracts of *Dracocephalum* and selected phenolics and water-soluble polysaccharides were investigated via radical-scavenging and ferrous (II) ion chelating assays. This paper demonstrates that herbs and roots of *D. austriacum* and *D. botryoides* are rich sources of metabolites and could be valuable plants for new biologically active products. To the best of our knowledge, this is the first study of whole plant metabolites and their antioxidant activity in *D. austriacum* and *D. botryoides*.

## 1. Introduction

For thousands of years, medicinal plants have been a valuable source of therapeutic agents, and they remain an important basis for the discovery of modern medicines [1]. The practice of herbal medicine builds on indigenous knowledge of the use of native plants for both the prevention and treatment of diseases. Local people have developed effective methods for identifying, collecting, using, and conserving medicinal plants and their habitats [2]. The implementation of ethnopharmacological and phytochemical studies on the study of plants from the local flora is an important task in the search for promising medicinal raw materials [3].

**Figure 1:**
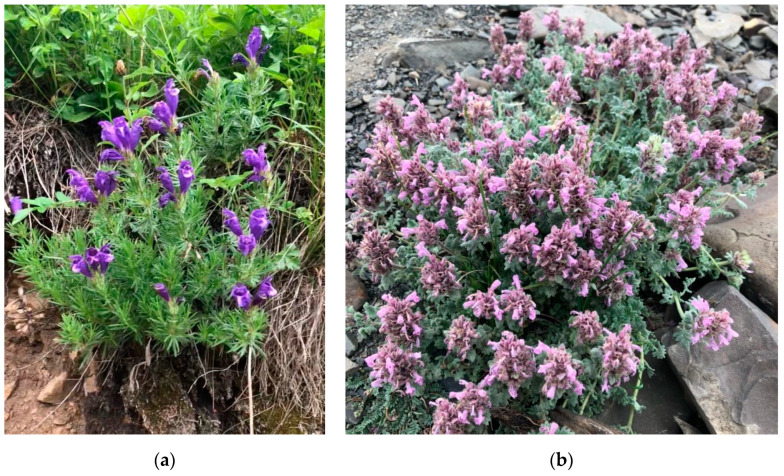
*Dracocephalum austriacum* (**a**) and *D. botryoides* (**b**) in their natural habitat in the flowering stage (Azerbaijan, Gryz, Guba region).

The Caucasus is a floristically diverse region and is of interest to modern researchers due to its extensive thickets of medicinal plants and the widespread use of local plant resources as medicinal plant raw materials [4]. Plants of the Lamiaceae family, and the *Dracocephalum* genus (dragonhead) in particular, are among the most used in folk medicine in the Caucasus [5,6]. There are 74 species in the *Dracocephalum* genus [7], with about 30 species of economic significance and used as medicinal plants [8]. This genus is of interest to researchers due to its wide range of biological activities, such as anti-inflammatory [9,10], antibacterial [11], antioxidant [12], etc. The genus *Dracocephalum* is represented in the Caucasus, in particular Azerbaijan, by six species, among which *Dracocephalum botryoides* Steven and *Dracocephalum austriacum* L. are of special interest as they have not been investigated (Figure 1) [13]. *Dracocephalum botryoides* is a perennial plant with numerous simple and branched pubescent stems, 10–20 cm in height; ovate-rounded leaves, 1–1.5 cm long and 0.8–1.2 cm wide; and purple flowers on short pedicels with false whorls. It grows on rocky slopes, near streams and on rocks in the alpine belt, at an altitude of 2500–3600 m. *Dracocephalum austriacum* is a perennial plant with single or several pubescent stems, 20–60 cm in height; linear or lanceolate leaves, 2–3 cm long and 1–2.5 mm wide; and dark purple flowers on short pedicels with false whorls. It grows on limestone and rocky slopes, on steppe and subalpine meadows up to 2400 m [14].

Freshly harvested *D. austriacum* and *D. botryoides* are applied as a spice in Azerbaijani cuisine, and the fresh herb of *D. botryoides* is used in vegetable salads. These *Dracocephalum* species are used in the preparation of the famous Azerbaijani dish *kükü*, which is made by some local people from eggs, fresh herbs, butter, and milk [15]. Moreover, one of the features of the first dishes of Azerbaijani cuisine is their use for medicinal purposes. Thus, *xəmiraşı* flour soup, with the addition of dragonhead, was used in Azerbaijani villages in ancient times to treat diseases of the respiratory and gastrointestinal tracts [16]. Furthermore, an herb infusion of *D. botryoides* was applied in liver diseases, gastritis, and ulcers, while an herb infusion of *D. austriacum* was employed as an anti-inflammatory and wound healing remedy in Azerbaijani folk medicine [17].

There is information about the essential oils for both *Dracocephalum* species [18] and germacrone for *D. botryoides* [19]. However, the chemical data known about the *Dracocephalum* genus makes it possible to characterize some species of this genus as known and valuable sources of bioactive components. Earlier phytochemical studies of the genus *Dracocephalum* reported the presence of flavonoids, terpenoids, alkaloids, lignans, coumarins, polysaccharides, and cyanogenic glycosides [9,20]. An increased interest in phenolic compounds was noted when analysing chemical information on various *Dracocephalum* species, which can be explained by their good antioxidant activity [12,21,22,23].

As part of the ongoing work involving the metabolomic study of the *Dracocephalum* genus [24,25,26,27], we realized the first analysis of herb and root extracts of *D. austriacum* and *D. botryoides* using high-performance liquid chromatography with diode array and electrospray triple quadrupole mass detection (HPLC-PDA-ESI-QQQ-MS). Water-soluble polysaccharides (WSPS) of the herb and roots of *D. austriacum* and *D. botryoides* were also investigated. As most of the metabolites found in *D. austriacum* and *D. botryoides* were phenolic compounds, the antioxidant potential of the studied extracts of *Dracocephalum*, as well as WSPS and selected phenolic compounds, was studied using five in vitro models. To the best of our knowledge, this is the first study of whole plants metabolites and their antioxidant activity for *D. austriacum* and *D. botryoides*.

## 2. Results and Discussion

### 2.1. LC-MS Profiles of Herb and Root Extracts of Two Dracocephalum Species

**Figure 2:**
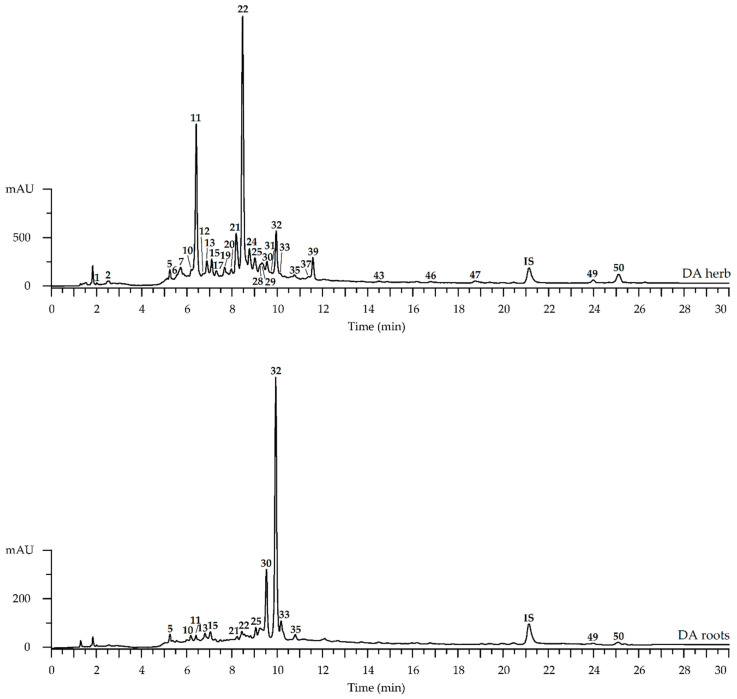
High-performance liquid chromatography with photodiode array detection (HPLC-PDA) chromatograms (270 nm) of herb and root extracts of *Dracocephalum austriacum* (DA). Compounds are numbered as listed in Table 1. IS—3′,4′-di-*O*-acetyl-cis-khellactone (5 μg/mL).

**Figure 3:**
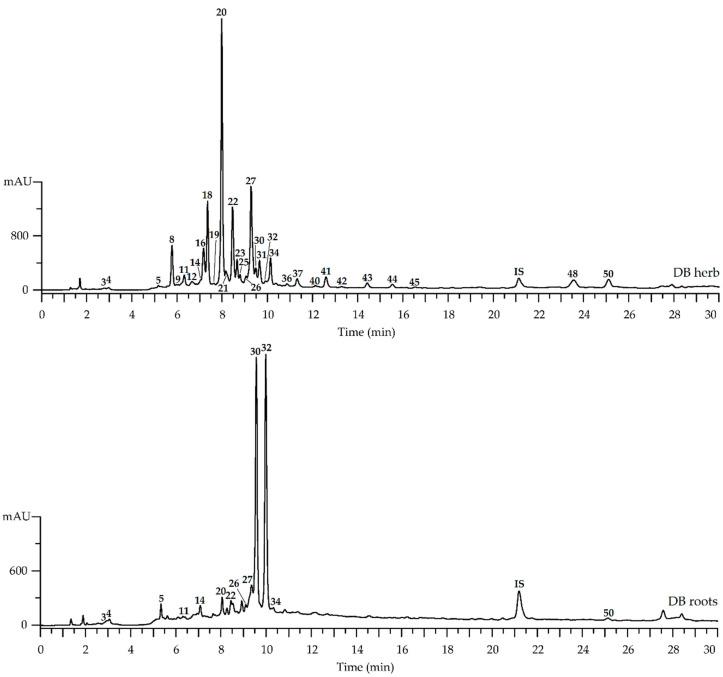
High-performance liquid chromatography with photodiode array detection (HPLC-PDA) chromatograms (270 nm) of herb and root extracts of *Dracocephalum botryoides* (DB). Compounds are numbered as listed in Table 1. IS—3′,4′-di-*O*-acetyl-cis-khellactone (5 μg/mL).

Chromatographic profiles of herbs and roots of *D. botryoides* and *D. austriacum* were realized by high-performance liquid chromatography with photodiode array and electrospray ionization mass spectrometric detection (HPLC-PDA-ESI-QQQ-MS). Compounds of both *Dracocephalum* species were identified after a precise interpretation of the chromatographic and spectral data (using retention times and ultraviolet-visible spectra/mass spectral patterns, respectively) in comparison with reference standards and literature data. The obtained HPLC-PDA-ESI-QQQ-MS chromatograms of herb and root extracts from *D. austriacum* and *D. botryoides* revealed the presence of 50 compounds with interpretable data (Figure 2 and Figure 3), details of which are shown in Table 1.

#### 2.1.1. Benzoic Acid Derivatives, Phenylpropanoids and Lignans

Two derivatives of benzoic acid {4-hydroxybenzoic acid 4-*O*-glucoside (**8**), 4-hydroxybenzoic acid *O*-hexoside-*O*-malonyl ester (**16**)} were found in the herb of *D. botryoides*. 4-Hydroxybenzoic acid 4-*O*-glucoside (**8**) was identified using the reference standard. The mass spectrometric analysis of compound **16** demonstrated the loss of malonyl (86 a.m.u.) and hexose fragments (162 a.m.u.), and the remaining fragment with *m*/*z* 299 was related to the 4-hydroxybenzoic acid *O*-hexoside moiety. The assumed structure of compound **16** was found to be 4-hydroxybenzoic acid *O*-hexoside-*O*-malonyl ester. Compound **8** has not been found previously in the *Dracocephalum* genus but has been detected in *Nandina domestica* before [28].

Seventeen compounds were determined as phenylpropanoids, separated into danshensu (**3**) and its derivatives {danshensu *O*-hexoside (**1**) and danshensu *O*-acetyl ester (**4**)}; caffeic (**15**) and quinic acid derivatives {caftaric acid (**2**), 4-*O*-caffeoylquinic acid (**5**), 5-*O*-caffeoylquinic acid (**11**), 3-*O*-caffeoylquinic acid (**12**), caffeic acid *O*-hexoside (**13**) and 1,3-di-*O*-caffeoylquinic acid (**14**)}, derivatives of *p*-coumaric acid {benzyl *O*-*p*-coumaroyl-*O*-desoxyhexoside-*O*-hexoside (**38**) and benzyl *O*-*p*-coumaroyl-*O*-hexoside (**39**)} and derivatives of rosmarinic acid (**30**) {lithospermic acid B *O*-hexoside (**31**), rosmarinic acid di-*O*-methyl ester (**47**), lithospermic acid A (**33**) and lithospermic acid B (**32**)}. The presence of danshensu (**3**) in the herb and roots of *D. botryoides* was established by comparison with the reference standard. The mass spectrometric analysis of **1** and **4** demonstrated the loss of a carbohydrate fragment of hexose (162 a.m.u.) and an acetyl fragment (42 a.m.u.), leaving the fragment with *m*/*z* 197, related to the danshensu moiety. The provisional structures of **1** and **4** were found to be danshensu *O*-hexoside and danshensu *O*-acetyl ester, respectively. Trace amounts of danshensu *O*-hexoside (**1**) was revealed in the herb of *D. austriacum*, while danshensu *O*-acetyl ester was detected in the roots of *D. botryoides*. The presence of danshensu and its derivatives in *Dracocephalum* species has been revealed for the first time; previously, danshensu was detected in the genus *Salvia* [29] and *Orthosiphon* [30].

The identification of caffeic (**15**) and quinic acids derivatives, such as monocaffeoylated 3-*O*-(**12**), 4-*O*-(**5**), 5-*O*-(**11**) and dicaffeoylated 1,3-di-*O*-caffeoylquinic acids (**14**), was carried out by comparing the retention times, UV- and mass-spectra with reference standards. The authentication of caftaric acid (**2**), a derivative of caffeic and tartaric acids, was realised in the same way. Component **13** produced a deprotonated ion with *m*/*z* 341 and a dehexosylated fragment with *m*/*z* 179, characteristic for caffeic acid *O*-hexoside. Caffeic acid (**15**) and its hexoside (**13**) were revealed in the herb and roots of *D. austriacum*. Caftaric acid (**2**) was also detected in the herb of *D. austriacum*. Monocaffeoylated 3-*O*-(**12**), 4-*O*-(**5**) and 5-*O*-caffeoylquinic acids (**11**) were revealed in both investigated *Dracocephalum* species, while 1,3-di-*O*-caffeoylquinic acid (**14**) was found in trace amounts in the herb and roots of *D. botryoides* only. Caffeic acid derivatives are characteristic compounds for *Dracocephalum* species. Caffeic acid (**15**) was previously revealed in *D. moldavica* [21], *D. peregrinum* [31], *D. ruyschiana* [32] and *D. palmatum* [24]. 3-*O*-caffeoylquinic acid (**12**) was discovered in *D. peregrinum* [31], *D. tanguticum* [33] and *D. palmatum* [24], while 5-*O*-caffeoylquinic acid (**11**) was previously found in *D. palmatum* [24]. Caftaric acid was also detected earlier in *D. palmatum* [24]. There are no literature data on the early detection of 4-*O*-caffeoylquinic acid (**5**) in other species of the *Dracocephalum* genus.

Rosmarinic acid (**30**) and its derivatives, lithospermic acid A (**33**) and lithospermic acid B (salvianolic acid B, **32**), were identified by comparing their retention times and UV- and mass spectrometric data with reference standards. Derivatives of rosmarinic acid were described as lithospermic acid B *O*-hexoside (**31**) and rosmarinic acid di-*O*-methyl ester (**47**). Compounds **30**–**32** were revealed in both investigated *Dracocephalum* species, while components **33** and **47** were detected only in *D. austriacum*. Rosmarinic acid (**30**) has previously been found in *D. moldavica* [21], *D. kotschyi* [34], *D. palmatum* [25] and *D. forrestii* [35]. Lithospermic acid B (**32**) was previously detected in *D. forrestii* [35] and *D. palmatum* [25], while lithospermic acid A (**33**) was revealed in *D. forrestii* [36].

In the mass spectra of compounds **38** and **39**, we found the primary loss of the neutral fragment with *m*/*z* 146, related to the *p*-coumaroyl moiety (*m*/*z* 561 [M-H]^−^ → 415; *m*/*z* 415 [M-H]^−^ → 269), and the remaining fragment with *m*/*z* 269 was related to the benzoyl-hexose. The provisional structures of **38** and **39** were found to be benzyl *O*-*p*-coumaroyl-*O*-desoxyhexoside-*O*-hexoside and benzyl *O*-*p*-coumaroyl-*O*-hexoside, respectively. Both compounds were detected in the herb of *D. austriacum*.

Four lignans {schizotenuin A (**46**), nepetamultin A *O*-hexosides (**48**, **49**) and nepetamultin A (**50**)} were detected in the herbs and roots of *D. austriacum* and *D. botryoides*. Schizotenuin A (**46**) was identified using the reference standard and was revealed in the herb of *D. austriacum* in trace amounts. Benzofuran lignan nepetamultin A (**50**) was identified in the herbs and roots of *D. austriacum* and *D. botryoides* by comparison with the reference standard. Compounds **48** and **49** had UV spectra typical of caffeic acid derivatives (λ_max_ 290 and 321 nm), and their mass spectra showed the primary loss of the particle with *m*/*z* 162, related to hexose, and the remaining fragment, with *m*/*z* 743, was related to nepetamultin A and the loss of the particle with *m*/*z* 212 due to the loss of hydroxyhydrocafeic acid. The supposed structures of **49** and **50** were found to be nepetamultin A *O*-hexosides. Compounds **46** and **50** have not previously been found in the *Dracocephalum* genus, although schizotenuin A has been detected in *Lycopus lucidus* [37] and *Schizonepeta tenuifolia* [38], while nepetamultin A has been revealed in *Nepeta multifida* [39].

#### 2.1.2. Flavonoids

Twenty-seven flavonoid compounds were determined in herbs and roots of *D. austriacum* and *D. botryoides*, belonging to one of five groups, depending on their aglycone structure: flavones luteolin (12 components), apigenin (6 components) and acacetin (3 components), and flavanones eriodictyol (3 components) and naringenin (3 components). The luteolin derivatives were the largest group with 12 compounds including non-acylated and acylated derivatives. Seven non-acylated compounds were identified using the reference standards as luteolin 7,4′-di-*O*-rutinoside (**10**), luteolin 7-*O*-rutinoside-4′-*O*-glucoside (cynarotriside, **17**), luteolin 7-*O*-rutinoside (scolymoside, **19**), luteolin 7-*O*-glucoside (cynaroside, **20**), luteolin 4′-*O*-glucoside (**22**), luteolin 3′-*O*-glucoside (**24**) and luteolin (**37**). The most diverse composition of non-acylated luteolin derivatives was noted in the herb of *D. austriacum* (**10**, **17**, **19**, **20**, **22**, **24** and **37**), while only two compounds (**10** and **22**) were found in the roots. Compounds **37**, **19**, **20** and **22** were present in the herb of *D. botryoides*, while compounds **20** and **22** were detected in the roots of this species. The remaining compounds gave an aglycone ion with *m*/*z* 285 in the mass spectra, and the loss of hexose fragments (*m*/*z* 162) and desoxyhexose (*m*/*z* 146) was observed in a 3:2 ratio (luteolin tri-*O*-hexoside-di-*O*-desoxyhexoside, **6** and **7**) and in a 1:1 ratio (luteolin *O*-desoxyhexoside-*O*-hexoside, **21**). Components **6** and **7** were detected in the herb of *D. austriacum*, while **21** was revealed in herb and roots of *D. austriacum* and in the herb of *D. botryoides*. The acylated luteolin derivative luteolin 7-*O*-(6″-*O*-acetyl)-glucoside (**27**) was identified by comparison with the reference standard. Compound **34** gave an aglycone ion with *m*/*z* 285 in the mass spectra, and the consistent loss of particles with *m*/*z* 42, related to acetyl, and the loss of a hexose fragment (*m*/*z* 162) was observed. The provisional structure of **34** was found to be luteolin 7-*O*-(2″,6″-di-*O*-acetyl)-hexoside. Compounds **27** and **34** were revealed in the herb and roots of *D. botryoides*. Luteolin was previously discovered in *D. moldavica* [39], *D. peregrinum* [31], *D. kotschyi* [22], *D. subcapitatum* [40], *D. rupestre* [41], *D. tanguticum* [42] and *D. palmatum* [25]. Two rare di-*O*-glucosides of luteolin, **10** and **17,** were previously found in *D. palmatum* [24], while luteolin 7-*O*-rutinoside was previously detected in *D. peregrinum* [31] and *D. palmatum* [24]. Luteolin 7-*O*-glucoside was previously revealed in *D. rupestre* [43], *D. peregrinum* [31], *D. moldavica* [21], *D. kotschyi* [44], *D. tanguticum* [42] and *D. palmatum* [25], while compound **22** was previously detected in *D. palmatum* [24]. Luteolin 3′-*O*-glucoside has been found in *D. thumiflorum* [45] and *D. nutans* [46], while the acylated luteolin derivative **27** was previously discovered in *D. palmatum* [27].

The flavanone eriodictyol (**36**) and its derivatives eriodictyol 7-*O*-rutinoside (**9**) and eriodictyol 7-*O*-glucoside (**18**) were identified by comparing their retention times, UV and ESI-MS patterns with reference standards. Compounds **9**, **18** and **36** were found in the herb of *D. botryoides*. Eriodictyol was previously identified in *D. peregrinum* [31], *D. palmatum* [25], *D. rupestre* [47] and *D. tanguticum* [42]; eriodictyol 7-*O*-rutinoside (**9**) was revealed in *D. palmatum* [24], while eriodictyol 7-*O*-glucoside (**18**) was detected in *D. rupestre* [9] and *D. palmatum* [25]. The identification of the flavanone naringenin (**40**) and its derivatives naringenin 7-*O*-rutinoside (**23**) and naringenin 7-*O*-glucoside (**26**) was carried out by comparison of retention times, UV- and mass-spectra with reference compounds. Naringenin 7-*O*-rutinoside and naringenin were detected in the herb of *D. botryoides*, while naringenin 7-*O*-glucoside was found in both the herb and roots of this species. Compounds **40** and **26** were previously revealed in *D. rupestre* [9] and *D. palmatum* [25], while compound **23** was discovered in *D. forrestii* [48].

The apigenin group of flavones consisted of non-acylated and acylated derivatives. After comparison with reference standards, the non-acylated compounds were identified as apigenin (**41**), apigenin 7-*O*-rutinoside (**25**) and apigenin 7-*O*-glucoside (**28**). Apigenin was revealed in the herb of *D. botryoides*, and apigenin 7-*O*-rutinoside was detected in the herb and roots of *D. austriacum*, while apigenin 7-*O*-glucoside was found only in the herb of this species. The acylated apigenin derivatives apigenin 7-*O*-(4″-malonyl-6″-*O*-acetyl)-glucoside (**42**) and apigenin 7-*O*-(6″-*O*-acetyl)-glucoside (**44**) were identified by comparison with the reference standard. Compound **45** gave an aglycone ion with *m*/*z* 269 in the mass spectra, and the consistent loss of particles with *m/z* 42, related to acetyl, and the loss of a hexose fragment (*m*/*z* 162) was observed. The provisional structure of compound **45** was found to be apigenin *O*-hexoside-di-*O*-acetyl ester. All acylated apigenin derivatives (**42**, **44** and **45**) were revealed in the herb of *D. botryoides*. Apigenin was previously discovered in *D. rupestre* [9], *D. moldavica* [49], *D. kotschyi* [10], *D. palmatum* [25], *D. polychaetum* [50] and *D. oligadenium* [51], and apigenin 7-*O*-rutinoside has been detected in *D. heterophyllum* [52] and *D. palmatum* [24]. Apigenin 7-*O*-glucoside was found previously in *D. kotschyi* [53], *D. palmatum* [25], *D. tanguticum* [54] and *D. moldavica* [55], and apigenin 7-*O*-(6″-*O*-acetyl)-glucoside was previously revealed in *D. palmatum* [27], while apigenin 7-*O*-(4″-malonyl-6″-*O*-acetyl)-glucoside has not previously been found in any species of the genus *Dracocephalum* but has been identified in *Matricaria chamomilla* [56].

The acacetin group of flavones was represented by three compounds. Acacetin 7-*O*-glucoside (**43**) was identified by comparing its retention time, UV and ESI-MS pattern with the reference standard. The remaining compounds showed the loss of hexose fragments with *m*/*z* 162 or fragments of desoxyhexose (*m*/*z* 146) in ratios of 3:1 (acacetin tri-*O*-hexoside-*O*-desoxyhexoside, **29**) and 1:1 (acacetin *O*-desohyhexoside-*O*-hexoside, **35**) in the mass spectra. Compound **35** was found in the herb and roots of *D. austriacum*, while compound **29** was found only in the herb of *D. austriacum*. Meanwhile, compound **43** was detected in herbs of both *D. austriacum* and *D. botryoides*. Acacetin 7-*O*-glucoside was previously identified in *D. foetidum* [57], *D. tanguticum* [58], *D. moldavica* [58], *D. peregrinum* [31], *D. kotschyi* [53] and *D. palmatum* [24].

As a result of the chromatographic research of *D. austriacum* and *D. botryoides*, 50 metabolites of various chemical groups were identified. This study demonstrates that the whole plant of both *Dracocephalum* species are characterized by a specific accumulation of phenolic metabolites. The highest content of phenolic compounds was typically found in the herb of both *Dracocephalum* species, while the roots were characterized by the lowest phenolic diversity. Flavonoid derivatives of luteolin and eriodictyol (luteolin 7-*O*-glucoside, luteolin 7-*O*-(6″-*O*-acetyl)-glucoside, luteolin 4′-*O*-glucoside and eriodictyol 7-*O*-glucoside) were the main compounds in herb of *D. botryoides*, while luteolin 4′-*O*-glucoside and 5-*O*-caffeoylquinic acid dominated in herb of *D. austriacum*. The roots of *D. austriacum* and *D. botryoides* had a similar chemical profile, with a predominance of the phenylpropanoids rosmarinic acid and lithospermic acid B. This may affect the biological properties of these species, particularly the antioxidant activity of dragonhead extracts, since the activity of diverse phenolic groups is known to be variable [59,60,61].

**Table 1:** Retention times (t), ultraviolet (UV), and mass spectral (ESI-MS) data of compounds **1**–**50** were found in leaves and roots of *D. austriacum* and *D. botryoides*, in addition to their content (mg/g of dry plant weight, in brackets S.D.).

No	t, min	Compound [Ref.]	Ident. Level ^a^	UV Spectrum, λ_max_, nm	ESI-MS, [M-H]^−^, *m*/*z*	ESI-MS, Additional Ions, *m*/*z*	*D. austriacum*		*D. botryoides*	
							Herb	Roots	Herb	Roots
**1**	2.00	Danshensu *O*-hexoside	2	280	359	197, 179, 135	trace ^b^			
**2**	2.52	Caftaric acid	1	328	311	179, 149, 135	0.14 (0.00)			
**3**	2.78	Danshensu	1	280	197	179, 135			trace	trace
**4**	3.08	Danshensu *O*-acetyl ester	2	280	239	197, 179, 135			trace	trace
**5**	5.28	4-*O*-Caffeoylquinic acid	1	328	353	191, 179, 173, 135	0.58 (0.02)	0.14 (0.00)	trace	1.02 (0.04)
**6**	5.51	Luteolin tri-*O*-hexoside- di-*O*-desoxyhexoside	2	263, 333	1063	901, 755, 593, 447, 285	trace			
**7**	5.62	Luteolin tri-*O*-hexoside- di-*O*-desoxyhexoside	2	263, 333	1063	901, 755, 593, 447, 285	0.08 (0.00)			
**8**	5.83	4-Hydroxybenzoic acid 4-*O*-glucoside	1	256	299	137			2.43 (0.10)	
**9**	5.98	Eriodictyol 7-*O*-rutinoside (eriocitrin)	1	287	595	449, 287			trace	
**10**	6.22	Luteolin 7, 4′-di-*O*-rutinoside	1	265, 333	901	755, 593, 447, 285	trace	0.11 (0.00)		
**11**	6.42	5-*O*-Caffeoylquinic acid	1	328	353	191, 165	3.14 (0.12)	0.09 (0.00)	0.52 (0.02)	trace
**12**	6.74	3-*O*-Caffeoylquinic acid	1	328	353	191, 179, 135	trace		0.08 (0.00)	trace
**13**	6.81	Caffeic acid *O*-hexoside	2	328	341	179, 135	0.79 (0.03)	0.33 (0.02)		
**14**	7.00	1,3-Di-*O*-Caffeoylquinic acid	1	328	515	353, 191, 179, 135			trace	trace
**15**	7.09	Caffeic acid	1	327	179	135	0.47 (0.03)	0.58 (0.02)		
**16**	7.11	4-Hydroxybenzoic acid *O*-hexoside-*O*-malonyl ester	2	256	385	299, 137			1.80 (0.07)	
**17**	7.31	Luteolin 7-*O*-rutinoside-4′-*O*-glucoside (cynarotriside)	1	265, 334	755	593, 447, 285	0.11 (0.00)			
**18**	7.47	Eriodictyol 7-*O*-glucoside	1	287	449	287			4.83 (0.24)	
**19**	7.72	Luteolin 7-*O*-rutinoside (scolymoside)	1	255, 346	593	447, 285	0.14 (0.00)		0.06 (0.00)	
**20**	7.98	Luteolin 7-*O*-glucoside (cynaroside)	1	256, 345	447	285	0.09 (0.00)		22.14 (0.92)	1.29 (0.06)
**21**	8.21	Luteolin *O*-desoxyhexoside-*O*-hexoside	2	269, 337	593	447, 285	1.73 (0.07)	trace	0.15 (0.00)	
**22**	8.51	Luteolin 4′-*O*-glucoside	1	269, 337	447	285	18.36 (0.73)	trace	3.79 (0.15)	0.91 (0.03)
**23**	8.71	Naringenin 7-*O*-rutinoside (narirutin)	1	289	579	433, 271			1.47 (0.06)	
**24**	8.82	Luteolin 3′-*O*-glucoside	1	268, 342	447	285	1.20 (0.05)			
**25**	9.01	Apigenin 7-*O*-rutinoside	1	265, 335	577	431, 269	0.67 (0.03)	0.93 (0.04)		
**26**	9.04	Naringenin 7-*O*-glucoside	1	289	433	271			0.58 (0.02)	trace
**27**	9.24	Luteolin 7-*O*-(6″-*O*-acetyl)-glucoside	1	256, 344	489	447, 285			4.22 (0.17)	2.46 (0.10)
**28**	9.28	Apigenin 7-*O*-glucoside	1	266, 336	431	269	trace			
**29**	9.31	Acacetin tri-*O*-hexoside- *O*-desoxyhexoside	2	268, 333	915	445, 283	trace			
**30**	9.53	Rosmarinic acid	1	329	359	719, 395, 179, 161	0.98 (0.04)	7.39 (0.27)	1.19 (0.04)	10.85 (0.44)
**31**	9.72	Lithospermic acid B *O*-hexoside	2	250, 290, 306, 330	879	717, 537, 519, 179	trace		1.29 (0.05)	
**32**	9.96	Lithospermic acid B	1	251, 289, 305, 329	717	537, 519, 179	1.27 (0.06)	12.73 (0.63)	0.07 (0.0)	11.39 (0.46)
**33**	10.04	Lithospermic acid A	1	253, 289, 310	537	493, 295	trace	1.68 (0.07)		
**34**	10.09	Luteolin 7-*O*-(2″,6″-di-*O*-acetyl)-hexoside	2	256, 344	531	489, 447, 285			1.04 (0.02)	trace
**35**	10.74	Acacetin *O*-desoxyhexoside-*O*-hexoside	2	268, 332	591	445, 283	trace	0.76 (0.02)		
**36**	10.91	Eriodictyol	1	287	287				trace	
**37**	11.48	Luteolin	1	256, 346	285		trace		0.04 (0.00)	
**38**	11.53	Benzyl *O*-*p*-coumaroyl-*O*-desoxyhexoside-*O*-hexoside	2	316	561	415, 269	0.83 (0.03)			
**39**	12.02	Benzyl *O*-*p*-coumaroyl-*O*-hexoside	2	316	415	269	trace			
**40**	12.06	Naringenin	1	289	271				trace	
**41**	12.58	Apigenin	1	266, 337	269				0.09 (0.0)	
**42**	13.31	Apigenin 7-*O*-(4″-malonyl-6″-*O*-acetyl)-glucoside	1	266, 335	559	431, 269			0.02 (0.00)	
**43**	14.48	Acacetin 7-*O*-glucoside	1	268, 335	445	283	trace		0.08 (0.00)	
**44**	15.52	Apigenin 7-*O*-(6″-*O*-acetyl)-glucoside	1	265, 336	473	431, 269			0.07 (0.00)	
**45**	16.50	Apigenin *O*-hexoside-di- *O*-acetyl ester	2	265, 336	515	431, 269			trace	
**46**	16.82	Schizotenuin A	1	289, 320	715	1431, 535, 357	trace			
**47**	18.69	Rosmarinic acid di-*O*-methyl ester	2	330	387	359, 179, 161	trace			
**48**	23.53	Nepetamultin A *O*-hexoside	2	290, 321	905	743, 531, 355			0.69 (0.02)	
**49**	24.00	Nepetamultin A *O*-hexoside	2	290, 321	905	743, 531, 355	0.14 (0.00)	trace		
**50**	25.08	Nepetamultin A	1	290, 320	743	531, 355	0.35 (0.02)	trace	0.52 (0.01)	trace

^a^ Identification levels: (1) identified compounds after comparison of UV, mass-spectral data, and retention time with reference standards; (2) putatively annotated compounds after comparison of UV and mass-spectral data with literature data [62]. ^b^ traces—<LOQ (limit of quantification).

### 2.2. Water-Soluble Polysaccharides of Herb and Roots of Two Dracocephalum Species

The fractions of WSPS were obtained from the herb and root samples of *D. austriacum* and *D. botryoides* by hot (90 °C) water extraction, followed by triple ethanol precipitation, deproteinization, ion-exchange resin and polyamide purification. The pure WSPS gave 0.6–1.2% yield (of dry plant weight) and were completely soluble in hot water (Table 2). The total carbohydrate content of the WSPS varied from 88.3% (WSPS of herb of *D. botryoides*) to 92.8% (WSPS of roots of *D. austriacum*), including a content of 25.3–49.2% uronic acids in herb WSPS and 11.2–12.0% uronic acids in root WSPS. All fractions were protein positive despite the deproteinization procedure used; the lowest protein level was 1.0% (WSPS of roots of *D. botryoides*) and the highest was 1.8% (WSPS of herb of *D. austriacum*). Only the root WSPS gave a positive iodine reaction, indicating the presence of starch-like polymers opposite negative resorcinol and Fehling’s reactions typical for fructans and mannans. The β-glucosyl Yariv reagent showed the precipitation of all four WSPS, indicating the formation of arabinogalactan-protein complexes. The monosaccharide composition study showed that herb WSPS had the highest content of galacturonic acid (23.6–48.0 mol%), arabinose (12.1–27.4 mol%) and galactose (16.2–20.5 mol%), and root WSPS had the highest content of arabinose (21.3–25.5 mol%), galactose (23.9–24.1 mol%) and glucose (22.6–25.6 mol%).

**Figure 4:**
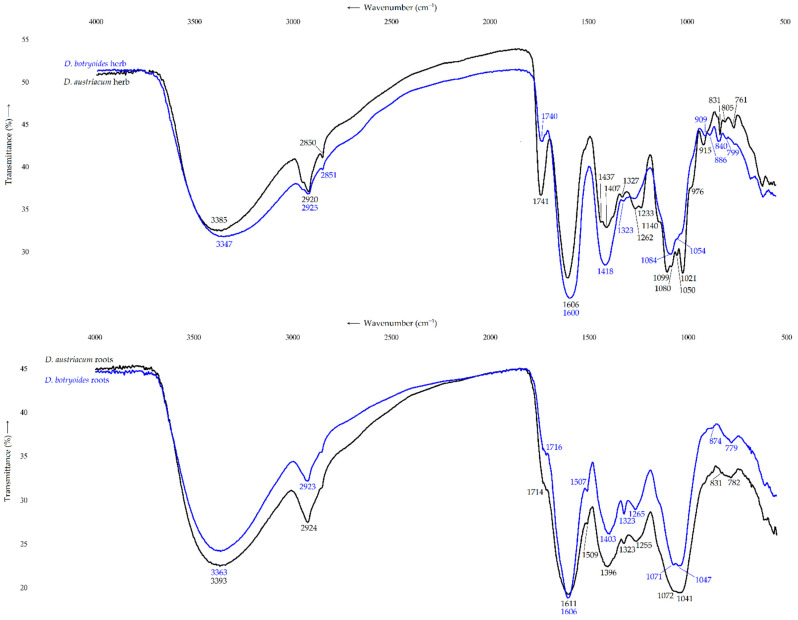
FTIR spectra of water-soluble polysaccharides of herbs and roots of *D. austriacum* and *D. botryoides*.

The Fourier-transform infrared (FTIR) spectral patterns of WSPS from herbs of *D. austriacum* and *D. botryoides* showed the typical pectin bands (Figure 4). There were intense bands of stretching vibrations of the esterified carboxylic groups (1714–1741 cm^−1^) and free carboxyls (1600–1606 cm^−1^), as well as bands of asymmetric and symmetric vibrations of the methyl esters (1437 cm^−1^), O-C-O fragments of esters (1260–1262, 1407–1418 cm^−1^) and C-H vibrations of the pyran rings (1327–1323 cm^−1^) [63]. The 900–1200 cm^−1^ region included vibration bands of the pyran rings and the glycoside bonds of pectins at 1140, 1099, 1080–1084, 1050–1054, 1021 and 976 cm^−1^ [64]. The presence of α-linkages and aldopyranoses were confirmed by the strong absorption bands at 831–840 cm^−1^ and 886 cm^−1^ in the anomeric region of the FTIR spectra, respectively. The FTIR spectral properties of WSPS from roots of *D. austriacum* and *D. botryoides* were generally close to that of the herb samples, except in the 900–1200 cm^−1^ region, and the anomeric region showed shapes specific for the starch-like polymers.

**Figure 5:**
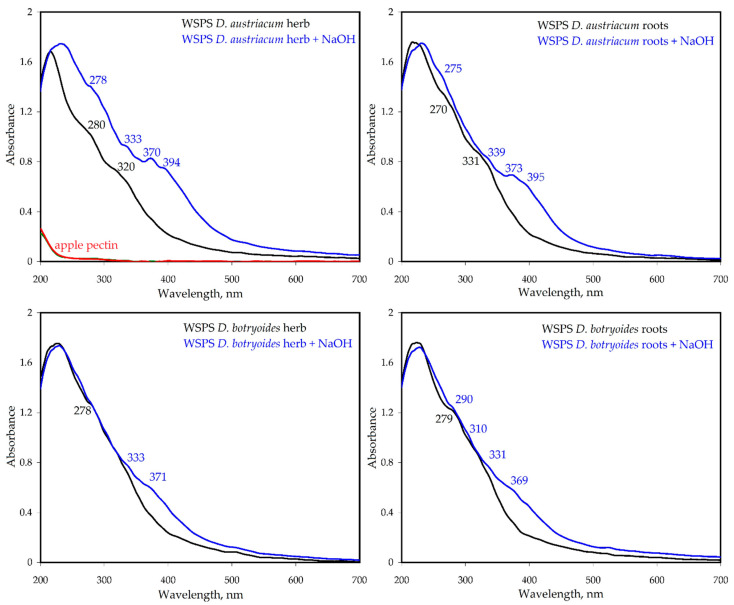
UV-Vis spectra of water-soluble polysaccharides of herbs and roots of *D. austriacum* and *D. botryoides*.

The spectra of WSPS in the ultraviolet region had shoulders at 270–280 nm and 320–331 nm, indicating the presence of phenolic fragments (Figure 5). The pure polysaccharides (like apple pectin) did not have maxima in the UV spectrum. The shortwave band was most likely the secondary benzoic band (B-band) caused by the *p*-hydroxy-phenyl fragments, while the longwave band was a K-band, owing to the n ⟶ π* transitions of the *p*-coumaroyl-like fragments. The ionization of phenolic functional groups by alkaline additives (NaOH) resulted in the appearance of additional maxima at 275–290, 310–339, 369–373 and 394–395 nm, typical for the simple phenolic compounds in alkaline media.

Alkaline hydrolysis of the WSPS of herbs and roots of *D. austriacum* and *D. botryoides* resulted in the release of 17 phenolic compounds, which were identified as benzoic acids, benzoic aldehydes and hydroxycinnamates using HPLC-DAD-MS (Table 3). The main phenolics in the WSPS of *D. austriacum* were *p*-methoxybenzoic acid (19.3–22.4%), vanillic acid (18.6–19.3%), vanillin (8.4–11.6%) and *p*-coumaric acid (10.7–15.2%), and the main phenolics in the WSPS of *D. botryoides* were *p*-methoxybenzoic acid (25.7–31.1%), vanillic acid (25.7–28.3%), veratric acid (14.7–19.7%) and vanillin (10.3–11.6%).

The WSPS of herbs and roots of *D. austriacum* and *D. botryoides*, therefore, had a complex structure consisting of at least three fragments of carbohydrate, protein, and phenolic nature. The polysaccharides were mostly pectins with additive arabinogalactan-protein complexes and starch-like compounds, linked with phenolic fragments of benzoic acids, benzoic aldehydes and hydroxycinnamates.

Dragonhead polysaccharides are still poorly researched; currently, there are only data relating to polysaccharides of the herb of *D. palmatum*, which are pectin, arabinogalactans, and a starch mixture [24], and information about polysaccharides of *D. moldavica* [20]. *Dracocephalum* membership in the subtribe Nepetinae, tribe Mentheae, which is included in the subfamily Nepetoideae of the Lamiaceae family, demonstrates the taxonomical proximity to *Agastache*, *Cedronella*, *Nepeta*, *Glechoma*, *Hymenocrater* and *Meehania* species [65]. Even so, polysaccharides have not been studied in the above genera. The closest known data about carbohydrate polymers belongs to the Menthinae and Salviinae subtribes, where galacturonans, glucans and arabinogalactans were found as components of *Lycopus* [66], *Mentha* [67,68], *Origanum* [69], *Prunella* [70], *Rosmarinus* [71], *Salvia* [72] and *Thymus* [73], indicating the prevalence of these polymers in the Mentheae tribe that *Dracocephalum* represents. The high phenolic content of polysaccharides of *D. austriacum* and *D. botryoides* seems unusual for Lamiaceae plants but is common for many other plants, showing the presence of cross-connected pectin-phenol and hemicellulose-lignin complexes [74]. Some of polyphenol linked polysaccharides are bioactive polymers with antioxidant, antidiabetic, and antitumor properties [75].

### 2.3. Antioxidant Activity of Extracts of D. austriacum and D. botryoides, Selected Phenolics and Water-Soluble Polysaccharides

Phenols are widely known as protective compounds against free radicals and toxic metals [76,77,78], so we decided to study the antioxidant potential of extracts of *D. austriacum* and *D. botryoides* as possible radical scavengers and metal chelators. Five well-known antioxidant assays were used to study the scavenging properties against 2,2-diphenyl-1-picrylhydrazyl radicals (DPPH^•^), 2,2′-azino-bis(3-ethylbenzothiazoline-6-sulfonic acid) cation radicals (ABTS^•+^), hydroxyl radicals and superoxide anion radicals, as well as the ferrous (II) ion chelating ability [79]. Seven selected compounds representing various phenolic groups detected in *D. austriacum* and *D. botryoides* were analysed, as well as WSPS. Trolox was used as a reference substance (Table 4).

The studied extracts of *Dracocephalum* demonstrated good scavenging effects against synthetic free radicals, such as DPPH^•^ and ABTS^•+^. The herb extract of *D. botryoides* was the most active, with IC_50_ values of 28.63 and 25.20 µg/mL for DPPH^•^ and ABTS^•+^ radicals, respectively, while roots of this species showed the lowest IC_50_ value among the extracts of *Dracocephalum* (40.67 and 36.14 µg/mL, respectively). Individual phenylpropanoids rosmarinic acid and lithospermic acid B showed superior scavenging activity against the DPPH^•^ radical, with IC_50_ values of 6.22 and 9.08 µg/mL, respectively, while the flavonoids eriodictyol 7-*O*-glucoside and luteolin 7-*O*-glucoside demonstrated maximal inhibition of the ABTS^•+^ radical (IC_50_ 7.39 and 8.26 µg/mL, respectively). The WSPS in *Dracocephalum* scavenged DPPH^•^ and ABTS^•+^ radicals with low efficiency (IC_50_ 102.85 and 82.79 µg/mL, respectively, for root WSPS *D. botryoides*). The same parameters for Trolox were 7.03 and 3.44 µg/mL, respectively. The ability of extracts of *Dracocephalum* to scavenge oxygen radicals (hydroxyl radicals and superoxide anion radicals) was moderate, with values prevailing for herb extract of *D. botryoides* (IC_50_ 103.28 and 85.67 µg/mL, respectively). Significant scavenging activity against OH^•^ radicals was found for luteolin 7-*O*-glucoside and luteolin 7-*O*-(6″-acetyl)-glucoside (53.29 and 55.73 µg/mL, respectively). Rosmarinic acid and lithospermic acid B demonstrated effective inactivation of O_2_^•−^ radicals (15.38 and 16.27 µg/mL, respectively). The potential of root WSPS from *D. botryoides* to scavenge OH^•^ and O_2_^•−^ radicals was good (IC_50_ 105.73 and 73.68 µg/mL, respectively). The maximum Fe^2+^-chelating ability was found with herb extract of *D. botryoides* (0.51 mM Fe^2+^-ions/g), while rosmarinic acid and eriodictyol 7-*O*-glucoside had the best chelating properties among the individual compounds (3.72 and 3.18 mM Fe^2+^-ions/g, respectively). The herb WSPS of *D. austriacum* demonstrated the best iron-chelating activity (4.31 mM Fe^2+^-ions/g). The same parameter for Trolox was 1.53 mM Fe^2+^-ions/g.

Previous studies have been conducted on the antioxidant activity of other *Dracocephalum* species. The water-soluble extract of *D. moldavica* revealed DPPH^•^ radical scavenging activity, with an IC_50_ of 445.90 μg/mL, and superoxide anion radical scavenging activity (IC_50_ 467.20 μg/mL) [21]. The methanol extract of *D. kotschyi* inhibited DPPH^•^, with IC_50_ values of 60.69 and 51.54 µg/mL for two-year and six-year-old samples, respectively [12]. The IC_50_ values for the DPPH^•^, ABTS^•+^ and OH^•^ radical-scavenging activities for the extract of *D. rupestre* were 50.01, 43.62 and 28.59 µg/mL, respectively [80], and the methanol extract of shoots of *D. polychaetum* had an IC_50_ value of 5600 µg/mL in the DPPH^•^ inhibiting assay [50].

The data obtained in five in vitro assays demonstrated a good efficacy for the herb extract of *D. botryoides* as an antioxidant agent. This trend is not surprising at all; the presence of strong antioxidants, such as phenylpropanoids and flavonoids in the herb extract of *D. botryoides* have led to such results. The antioxidant activity of herb and root extracts of *D. botryoides* and *D. austriacum*, as well as their water-soluble polysaccharides, have been shown here for the first time.

## 3. Conclusions

In this paper, the whole plants of *D. austriacum* and *D. botryoides* were shown to be natural accumulators of diverse metabolites of a phenolic and non-phenolic nature. The metabolites of these plant species were investigated for the first time using HPLC-PDA-ESI-QQQ-MS, and the water-soluble polysaccharides of roots of *D. austriacum* and *D. botryoides* were studied. The dragonhead polysaccharides were mainly found to be pectins with additive arabinogalactan-protein complexes and starch-like compounds, linked with phenolic fragments of benzoic acids, benzoic aldehydes and hydroxycinnamates. The phenolic diversity in herbs and roots of *D. austriacum* and *D. botryoides* implies the presence of antioxidant properties, which were confirmed by five in vitro assays (DPPH^•^, ABTS^•+^, OH^•^, O_2_^•−^ radical-scavenging activity and Fe^2+^-chelating activity). Thus, the information presented highlights the potential of the herbs and roots of *D. austriacum* and *D. botryoides* for possible future plant remedies or sources of new functional products.

## 4. Materials and Methods

### 4.1. Plant Material and Chemicals

Samples of *Dracocephalum austriacum* (herb and roots) were collected during the flowering period in the subalpine meadow in Azerbaijan in 5 locations, 10 samples from each near Gryz, Guba region (16.VI.2020, 41°21′93.2″ N, 48°24′55.8″ E, 1900 m a.s.l.; voucher No Az/LDR-0620/74-95). Samples of *D. botryoides* (herb and roots) were collected during the flowering period in alpine meadow in Azerbaijan in 5 locations, 10 samples from each near Khinalig, Guba region (5.VII.2020, 41°19′24.9″ N, 48°12′07.0″ E, 2850 m a.s.l.; voucher No Az/LDR-0720/87-114). The species were authenticated by authors Javanshir I. Isaev (Azerbaijan Medical University, Baku, Azerbaijan) and Daniil N. Olennikov (IGEB SB RAS, Ulan-Ude, Russia). Plant material was dried in the ventilated heat oven at 40 °C within 8–10 days and stored at 3–4 °C before analysis.

The reference compounds were purchased from BioCrick Co., Ltd. (Chengdu Tianfu, Sichuan, China); ChemFaces (Wuhan, Hubei, China); Extrasynthese (Lyon, France); PhytoLab GmbH & Co. KG (Vestenbergsgreuth, Germany); Sigma–Aldrich (St. Louis, MO, USA) or isolated in our laboratory (Table S1). Selected chemicals were from Biosupplies Australia Ply Ltd. (Victoria, Australia): Yariv reagent kit (Cat. No. 100-4); Sigma–Aldrich (St. Louis, MO, USA): acetonitrile for HPLC (Cat. No. 34851, ≥99.9%), anisic aldehyde (Cat. No. A88107, ≥98%), anthrone (Cat. No. 319899, ≥97%), arabinose (Cat. No. A3256, ≥99%), 2,2′-azino-bis(3-ethylbenzothiazoline-6-sulfonic acid) diammonium salt (Cat. No. A1888, ≥ 98%), *trans*-cinnamic acid (Cat. No. C80857, ≥99%), *p*-coumaric acid (Cat No. C9008, ≥98%), 3,5-dimethylphenol (Cat. No. 144134, ≥99%), 2,2-diphenyl-1-picrylhydrazyl radical (Cat. No. 281689, ≥97%), Fehling’s reagent I (Cat. No. 36018), *trans*-ferulic acid (Cat. No. 128708, ≥99%), ferrous sulphate (Cat. No. PHR1483), Folin–Ciocalteu′s phenol reagent (Cat No. 47641), formic acid (Cat. No. F0507, ≥95%), fucose (Cat. No. F8150, ≥98%), galactose (Cat. No. G0750, ≥99%), galacturonic acid monohydrate (Cat. No. 48280, ≥97%), gentisic acid (Cat. No. 149357, ≥98%), glucose (Cat. No. G8270, ≥99.5%), glucuronic acid (Cat. No. G5269, ≥98%), 4-hydroxybenzoic acid (Cat. No. H20059, ≥99%), isovanillic acid (Cat. No. 220108, ≥97%), *trans*-isoferulic acid (Cat. No. 05407, ≥98%), lithium perchlorate (Cat. No. 205281, ≥95%), mannose (Cat. No. M8574, ≥99%), *p*-methoxycinnamic acid (Cat. No. M13807, ≥99%), methanol (Cat. No. 322415, ≥99.8%), 4-methoxybenzoic acid (Cat. No. 117390, ≥99%), perchloric acid (Cat. No. 244252, ≥70%), protocatechuic acid (Cat. No. 03930590, ≥99%), rhamnose (Cat. No. W373011, ≥99%), resorcinol (Cat. No. 398047, ≥99%), ribose (Cat. No. R7500, ≥99%), sodium carbonate (Cat. No. S7795, ≥99%), trolox (Cat. No. 238813, ≥97%), vanillic acid (Cat. No. H36001, ≥97%), vanillin (Cat. No. V1104, ≥99%), veratric acid (Cat. No. D131806, ≥99%), syringic acid (Cat. No. S6881, ≥95%), xylose (Cat. No. X1500, ≥99%).

### 4.2. Plant Extracts Preparation

To prepare plant extracts for HPLC analysis, the ground plant material (particle size 0.125 μm; weight 200 mg) was treated with 70% methanol (2 mL) three times by sonication (ultrasonic bath, 30 min, 50 °C, ultrasound power 100 W, frequency 35 kHz). The obtained liquid extracts were centrifuged at 20 °C (6000× *g*, 10 min) and the supernatants were filtered through 0.22 μm syringe filters into the measuring flask (10 mL). The final volume was 10 mL with 70% methanol. The obtained methanol extracts were stored at 2 °C before treatment. Before analysis, internal standard (methanol solutions) was added to samples in a ratio of 1:1: herb extracts—3′,4′-di-*O*-acetyl-*cis*-khellactone (5 μg/mL), root extracts—3′,4′-di-*O*-acetyl-*cis*-khellactone (5 μg/mL).

The extracts of herbs and roots of *D. austriacum* and *D. botryoides* for the antioxidant study were prepared from the ground samples (50 g), treated by the 70% methanol (1 L) in an ultrasonic bath (30 min, 45 °C, ultrasound power 100 W, frequency 35 kHz), filtered, reduced in a vacuum until dryness and stored (−20 °C) before analysis. The yields of the extracts from *D. austriacum* were 23.4% for the herb sample, 19.8% for the root sample. The yields of the extracts from *D. botryoides* were 21.7% for the herb sample and 18.1% for the root sample.

### 4.3. High-Performance Liquid Chromatography with Photodiode Array Detection and Electrospray Ionization Triple Quadrupole Mass Spectrometric Detection (HPLC-PDA-ESI-QQQ-MS) Metabolite Profiling

High-performance liquid chromatography with photodiode array detection and electrospray ionization triple quadrupole mass spectrometric detection (HPLC-PDA-ESI-QQQ-MS) was used for the quantitative profiling of the extracts from *D. austriacum* and *D. botryoides*. Liquid chromatograph LC-20 Prominence coupled with photodiode array detector SPD-M30A (wavelength range 200–600 nm), triple-quadrupole mass spectrometer LCMS 8050 (all Shimadzu, Columbia, MD, USA) and GLC Mastro C18 column (2.1 × 150 mm, 3 μm; Shimadzu, Kyoto, Japan) was used. The two-component eluent system of eluent A (0.5% formic acid in water) and B (0.5% formic acid in acetonitrile) used the gradient program for the separation (%B): 0–4 min 4%, 4–6 min 4–10%, 6–9 min 10–15%, 9–15 min 15–25%, 15–20 min 25–38%, 20–25 min 38–70%, 25–30 min 70–88%, 30–40 min 88–4%, 40–50 min 4%. The injection volume was 1 μL and the flow rate was 120 μL/min. The UV-Vis spectra were recorded by an SPD-M20A photodiode detector (spectral range 200–600 nm) equipped with a post-column derivatization reactor. The used temperature levels were 300 °C in the ESI interface, 250 °C in the desolvation line, and 400 °C in the heat block. The flow values were 3 L/min for the nebulizing gas (N_2_), 10 L/min for the heating gas (air), and 0.3 mL/min for the collision-induced dissociation gas (Ar). The source voltage of mass spectra was 3 kV and the collision energy −10–30 eV (negative ionization) by the scanning range of *m*/*z* 80–1900. The LC-MS system was managed by LabSolution’s workstation software equipped with the inner LC-MS library. The integrated analysis of retention time, ultraviolet and mass spectra data after comparison with the reference standards and literature data was used for the identification of metabolites [62].

### 4.4. HPLC-PDA-ESI-QQQ-MS Metabolite Quantification

To quantify 50 metabolites of herbs and roots of *D. austriacum* and *D. botryoides*, the HPLC-PDA-ESI-QQQ-MS conditions were used (Section 4.3). Totally, 34 reference standards were separately weighed (10 mg) and dissolved in the methanol-DMSO mixture (1:1) in volumetric flasks (10 mL) preparing the stock solution (1000 µg/mL) used for the calibration curve building. The calibration solution (1, 10, 25, 50, 100 µg/mL) chromatographed in known HPLC-PDA-ESI-QQQ-MS conditions and mass spectral data was used to create ‘concentration–mass spectrometric peak area’ correlation. The principal validation criteria including correlation coefficients (r^2^), standard deviation (S_YX_), limits of detection (LOD), limits of quantification (LOQ), and linear ranges were found using the known method [81] (Table S2). Five HPLC runs were sufficient for the quantitative analyses and the results were expressed as mean value ± standard deviation (S.D.).

### 4.5. Polysaccharides Extraction and Analysis

The samples of dried milled herb and roots of *D. austriacum* and *D. botryoides* (100 g) were added to distilled water (1.0 L), heated on a boiled water bath (1 h) and after cooling to room temperature water extracts were filtered under reduced pressure and concentrated down in vacuo to 200 mL. The concentrated residues were mixed with 95% ethanol (1:5) and after 2 h the precipitates were centrifuged at 3000× *g*. The crude polysaccharide fractions were redissolved in 200 mL of water. The Sevag method [82] was applied for deproteination and was followed by dialysis for 48 h against distilled water using dialysis tubes with an MW-cut off of 2 kDa (Sigma–Aldrich, St. Louis, MO, USA). The non-dialysed parts were loaded on to a KU-2-8 cation-exchange resin column (H^+^-form, 200 g; Closed Joint-Stock Company Tokem, Kemerovo, Russia) which were eluted with 2 L of distilled water. Eluates were concentrated in vacuo up to 200 mL and then liophylized. The resulting water-soluble polysaccharides were whitish powders.

For estimation of total carbohydrate content modified anthrone-H_2_SO_4_ spectrophotometric assay with glucose as a standard compound was used [83]. To evaluate content of uronic acids 3,5-dimethylphenol method was applied calculated as galacturonic acid [84]. The Bradford method using Coomassie G250 was employed for determination of protein content [85]. Phenolic content was analysed using the Folin–Ciocalteu method [86]. Reactions of water-soluble polysaccharides solutions with iodine, resorcinol, Yariv and Fehling’s reagents were carried out accordingly [87,88,89]. The IR spectra were registered in a spectral range of 4000–600 cm^−1^ using a FT-801 Fourier-transform infrared spectrometer (Simex, Novosibirsk, Russia) coupled with a single reflection ATR device. Alkaline hydrolysis procedure and HPLC-DAD-MS analysis conditions of the released products were as described by us earlier [90].

### 4.6. Antioxidant Activity

Microplate spectrophotometric assays were applied to investigate the scavenging activity of extracts of *D. austriacum* and *D. botryoides*, selected phenolics and water-soluble polysaccharides against 2,2-diphenyl-1-picrylhydrazyl radicals (DPPH^•^) [25], 2,2′-azino-bis(3-ethylbenzothiazoline-6-sulfonic acid) cation radicals (ABTS^•+^) [24], superoxide anion radicals (O_2_^•−^) [91], hydroxyl radicals (OH^•^) [91]. Ferrous (II) ion chelating activity was investigated by spectrophotometric assay [92]. Trolox was used as a reference standard (1–100 μg/mL in methanol). The results of DPPH^•^, ABTS^•+^, O_2_^•−^, OH^•^ assays were determined as IC_50_ (the half maximal inhibitory concentration) and calculated graphically using ‘concentration (μg/mL or mg/mL)–antioxidant activity (%)’ correlations. All the analyses were carried out five times, and the data are expressed as the mean value ± standard deviation (S.D.)

### 4.7. Statistical Analysis

Statistical analyses were performed by one-way analysis of variance, and the significance of the mean difference was determined by Duncan’s multiple range test. Differences at *p* < 0.05 were considered statistically significant. The results are presented as mean values ± standard deviations (S.D.). The linear regression analysis and generation of calibration graphs were conducted using Advanced Grapher 2.2 (Alentum Software Inc., Ramat-Gan, Israel).

## Figures and Tables

**Table 2:** Characteristics of water-soluble polysaccharides of *D. austriacum* and *D. botryoides* herb and roots.

Parameter	WSPS of *D. austriacum* Herb	WSPS of *D. austriacum* Roots	WSPS of *D. botryoides* Herb	WSPS of *D. botryoides* Roots
Yield, % ^1^	1.2	0.9	0.7	0.6
Carbohydrate content, % ^2^	90.5 ± 2.7	92.8 ± 2.4	88.3 ± 2.5	92.1 ± 2.8
Uronic acid content, % ^2^	49.2 ± 1.9	12.0 ± 0.4	25.3 ± 0.9	11.2 ± 0.4
Protein content, % ^2^	1.8 ± 0.0	1.7 ± 0.0	1.4 ± 0.0	1.0 ± 0.0
Phenolic content, % ^2^	3.3 ± 0.1	1.4 ± 0.0	4.0 ± 0.1	4.3 ± 0.1
Iodine reaction	negative	positive	negative	positive
Resorcinol reaction	negative	negative	negative	negative
Fehling’s reaction	negative	negative	negative	negative
β-Glucosyl Yariv reaction	positive	positive	positive	positive
Monosaccharide composition:				
Arabinose, mol%	12.1	21.3	27.4	25.5
Galactose, mol%	16.2	24.1	20.5	23.9
Glucose, mol%	6.3	22.6	13.4	25.6
Fucose, mol%	0.6	1.8	0.4	1.1
Mannose, mol%	3.0	8.3	4.4	7.7
Ribose, mol%	0.0	0.0	0.0	0.0
Rhamnose, mol%	10.8	6.4	8.1	3.8
Xylose, mol%	0.5	0.1	0.4	0.1
Galacturonic acid, mol%	48.0	12.6	23.6	10.0
Glucuronic acid, mol%	2.4	2.9	1.7	2.4

^1^ Percentage of dry plant weight. ^2^ Percentage of polysaccharide fraction weight.

**Table 3:** Phenolic compounds released after alkaline hydrolysis of water-soluble polysaccharides of herbs and roots of *D. austriacum* and *D. botryoides*.

Compound	Percentage of Compound, % of Total Phenolic Content			
	*D. austriacum* Herb	*D. austriacum* Roots	*D. botryoides* Herb	*D. botryoides* Roots
Benzoic acid	1.2 ± 0.0	2.5 ± 0.1	0.2 ± 0.0	0.1 ± 0.0
*p*-Hydroxybenzoic acid	1.1 ± 0.0	0.8 ± 0.0	0.1 ± 0.0	0.1 ± 0.0
*p*-Methoxybenzoic acid	19.3 ± 0.4	22.4 ± 0.5	31.1 ± 0.6	25.7 ± 0.5
Protocatechuic acid	0.5 ± 0.0	0.2 ± 0.0	0.1 ± 0.0	0.3 ± 0.0
Gentisic acid	0.2 ± 0.0	0.5 ± 0.0	0.5 ± 0.0	0.3 ± 0.0
Vanillic acid	18.6 ± 0.4	19.3 ± 0.4	25.7 ± 0.5	28.3 ± 0.6
Isovanillic acid	2.5 ± 0.0	1.4 ± 0.0	1.1 ± 0.0	0.9 ± 0.0
Veratric acid	7.2 ± 0.1	9.3 ± 0.2	14.7 ± 0.3	19.7 ± 0.4
Syringic acid	0.2 ± 0.0	3.4 ± 0.1	0.6 ± 0.0	1.4 ± 0.0
Anisic aldehyde	6.2 ± 0.1	7.2 ± 0.1	3.1 ± 0.1	2.2 ± 0.0
Vanillin	8.4 ± 0.1	11.6 ± 0.2	11.6 ± 0.2	10.3 ± 0.02
Cinnamic acid	2.6 ± 0.1	1.1 ± 0.0	0.3 ± 0.0	0.9 ± 0.0
*p*-Coumaric acid	15.2 ± 0.2	10.7 ± 0.2	2.5 ± 0.1	1.4 ± 0.0
*p*-Methoxycinnamic acid	9.2 ± 0.2	5.1 ± 0.1	3.9 ± 0.1	6.2 ± 0.1
Caffeic acid	0.7 ± 0.0	0.1 ± 0.0	0.0 ± 0.0	0.0 ± 0.0
Ferulic acid	3.8 ± 0.1	3.9 ± 0.1	3.6 ± 0.1	1.9 ± 0.0
Isoferulic acid	1.9 ± 0.0	0.4 ± 0.0	0.8 ± 0.0	0.2 ± 0.0

**Table 4:** Bioactivity of extracts of *D. austriacum* and *D. botryoides*, selected phenolics and water-soluble polysaccharides (WSPS) in five antioxidant assays ^a^.

Object	DPPH^•^^b^	ABTS^•^^+ b^	OH^• b^	O_2_^•^^− b^	FeCA ^c^
*D. austriacum* herb extract	37.15 ± 0.74 ^viii^	35.81 ± 0.88 ^v^	125.67 ± 3.79 ^vii^	108.26 ± 4.32 ^ix^	0.42 ± 0.02 ^iii^
*D. austriacum* root extract	35.19 ± 0.71 ^viii^	29.64 ± 0.72 ^iv^	139.09 ± 4.19 ^viii^	99.14 ± 3.82 ^ix^	0.38 ± 0.01 ^ii^
*D. botryoides* herb extract	28.63 ± 0.54 ^vii^	25.20 ± 0.50 ^iv^	103.28 ± 3.09 ^v^	85.67 ± 3.45 ^vii^	0.51 ± 0.02 ^iv^
*D. botryoides* root extract	40.67 ± 0.82 ^ix^	36.14 ± 0.93 ^v^	164.11 ± 4.99 ^ix^	135.24 ± 5.44 ^x^	0.27 ± 0.01 ^i^
5-*O*-Caffeoylquinic acid	7.63 ± 0.15 ^iii^	9.04 ± 0.17 ^iii^	68.25 ± 1.73 ^ii^	49.11 ± 1.47 ^ii^	2.28 ± 0.09 ^vii^
Luteolin 7-*O*-glucoside	9.20 ± 0.18 ^iv^	8.26 ± 0.15 ^ii^	53.29 ± 1.33 ^i^	60.83 ± 1.83 ^iv^	2.84 ± 0.11 ^ix^
Luteolin 7-*O*-(6″-acetyl)-glucoside	10.27 ± 0.22 ^v^	9.63 ± 0.20 ^iii^	55.73 ± 1.30 ^i^	67.09 ± 2.01 ^v^	2.42 ± 0.10 ^vii^
Luteolin 4′-*O*-glucoside	45.60 ± 0.87 ^x^	67.19 ± 1.28 ^vi^	123.14 ± 2.97 ^vii^	170.81 ± 5.18 ^xi^	1.65 ± 0.06 ^vi^
Eriodictyol 7-*O*-glucoside	12.04 ± 0.25 ^vi^	7.39 ± 0.14 ^i^	82.17 ± 2.05 ^iii^	53.02 ± 1.63 ^iii^	3.18 ± 0.12 ^ix^
Rosmarinic acid	6.22 ± 0.14 ^i^	25.73 ± 0.52 ^iv^	95.16 ± 2.47 ^iv^	15.38 ± 0.42 ^i^	3.72 ± 0.15 ^x^
Lithospermic acid B	9.08 ± 0.17 ^iv^	36.28 ± 0.71 ^v^	105.61 ± 2.50 ^v^	16.27 ± 0.49 ^i^	2.87 ± 0.12 ^ix^
*D. austriacum* herb WSPS	189.42 ± 3.78 ^xiii^	150.03 ± 3.75 ^ix^	197.83 ± 5.90 ^x^	89.57 ± 3.11 ^vii^	4.31 ± 0.18 ^xi^
*D. austriacum* root WSPS	>500	214.53 ± 5.35 ^x^	251.12 ± 7.53 ^xi^	129.11 ± 4.56 ^x^	1.19 ± 0.04 ^v^
*D. botryoides* herb WSPS	141.15 ± 2.80 ^xii^	105.63 ± 2.67 ^viii^	129.63 ± 3.80 ^vii^	82.56 ± 2.88 ^vii^	2.39 ± 0.09 ^vii^
*D. botryoides* root WSPS	102.85 ± 2.07 ^xi^	82.79 ± 2.06 ^vii^	105.73 ± 3.14 ^v^	73.68 ± 2.57 ^vi^	1.10 ± 0.05 ^v^
Trolox ^d^	7.03 ± 0.14 ^ii^	3.44 ± 0.69 ^v^	108.69 ± 0.04 ^v, vi^	165.14 ± 4.95 ^xi^	1.53 ± 0.06 ^vi^

^a^ DPPH^•^—2,2-diphenyl-1-picrylhydrazyl radical scavenging capacity; ABTS^•^^+^—2,2′-azino-bis(3-ethylbenzothiazoline-6-sulfonic acid) cation radical scavenging capacity; O_2_^•^^−^—superoxide radical scavenging capacity; OH^•^—hydroxyl radical scavenging capacity; FeCA—Fe^2+^-ions chelating activity. ^b^ IC_50_, μg/mL. ^c^ mM Fe^2+^-ions/g. ^d^ Reference compound. Averages ± standard deviation (S.D.) were obtained from five different experiments. Values with different numbers (i–xiii) in each column indicate statistically significant differences among groups at *p* < 0.05 by one-way ANOVA.

## Data Availability

Data are contained within the article.

## Supplementary Materials

The following are available online at https://www.mdpi.com/article/10.3390/plants11162126/s1. Table S1: Reference standards used for the qualitative and quantitative analysis by HPLC-PDA-ESI-QQQ-MS assays. Table S2: Regression equations, correlation coefficients, standard deviation, limits of detection, limits of quantification and linear ranges for 34 reference standards.
