# Supplementary material for: Caucasian Dragonheads: Phenolic Compounds, Polysaccharides, and Bioactivity of Dracocephalum austriacum and Dracocephalum botryoides
Source: Plants (Basel). 2022 Aug 15;11(16):2126. doi: 10.3390/plants11162126 (PMC9413935; doi:10.3390/plants11162126)
Supplement: Supplementary file 1 [file plants-11-02126-s001.zip › plants-1857234-supplementary.pdf]

plants

Supplementary Material

Caucasian Dragonheads: Phenolic Compounds, Polysaccharides, and Bioactivity of *Dracocephalum austriacum* and *Dracocephalum botryoides*

Nina I. Kashchenko[1,*], Gunay S. Jafarova [2], Javanshir I. Isaev [2], Daniil N. Olennikov [1], Nadezhda K. Chirikova [3]

[1] Laboratory of Medical and Biological Research, Institute of General and Experimental Biology, Siberian Division, Russian Academy of Science, 670047 Ulan-Ude, Russia; olennikovdn@mail.ru (D.N.O.)
[2] Department of Pharmacognosy, Azerbaijan Medical University, Anvar Gasimzade Street 14, Baku AZ1022, Azerbaijan; jafar.gunay.92@gmail.com (G.S.J.), isayev.cavanshir@amu.edu.az (J.I.I.)
[3] Department of Biochemistry and Biotechnology, North-Eastern Federal University, 58 Belinsky Street, 677027 Yakutsk, Russia; hofnung@mail.ru
* Correspondence: ninkk@mail.ru; Tel.: +8-983-421-73-40

Content

Table S1. Reference standards used for the qualitative and quantitative analysis by HPLC-PDA-ESI-QQQ-MS assays.

Table S2. Regression equations, correlation coefficients (r^2), standard deviation (S_{YX}), limits of detection (LOD), limits of quantification (LOQ) and linear ranges for 34 reference standards.

Table S1. Reference standards used for the qualitative and quantitative analysis by HPLC-PDA-ESI-QQQ-MS assays.

No in Table 1	Compound	Purity (≥), %	Manufacturer (Cat. No.) *	Used for Qualitative (QL) and Quantitative (QT) Analysis of Compounds (No in Table 1)
**2**	Caftaric acid	97	Sigma (15029)	QL: 2. QT: 2.
**3**	Danshensu	98	BioCrick (BCN8513)	QL: 3. QT: 1, 3, 4.
**5**	4-*O*-Caffeoylquinic acid	98	Sigma (65969)	QL: 5. QT: 5.
**8**	4-Hydroxybenzoic acid 4-*O*-glucoside	98	PlantMetaChem (C6201-H043)	QL: 8. QT: 8, 16.
**9**	Eriodictyol 7-*O*-rutinoside	98	Sigma (45714)	QL: 9. QT: 9.
**10**	Luteolin 7,4′-di-*O*-rutinoside	95	Lab collection/isolated from *D. palmatum* [26]	QL: 10. QT: 10.
**11**	5-*O*-Caffeoylquinic acid	95	Sigma (91213)	QL: 11. QT: 11.
**12**	3-*O*-Caffeoylquinic acid	95	Sigma (PHL89175)	QL: 12. QT: 12.
**14**	1,3-Di-*O*-caffeoylquinic acid	98	Sigma (D8196)	QL: 14. QT: 14.
**15**	Caffeic acid	98	Sigma (C0625)	QL: 15. QT: 13, 15.
**17**	Luteolin 7-*O*-rutinoside-4′-*O*-glucoside	95	Lab collection/isolated from *D. palmatum* [26]	QL: 17. QT: 17.
**18**	Eriodictyol 7-*O*-glucoside	99	Sigma (19474)	QL: 18. QT: 18.
**19**	Luteolin 7-*O*-rutinoside	95	Sigma (SMB00200)	QL: 19. QT: 19.
**20**	Luteolin 7-*O*-glucoside	98	Extrasynthese (1126 S)	QL: 20. QT: 6, 7, 20, 21, 34.
**22**	Luteolin 4′-*O*-glucoside	95	Extrasynthese (1412 S)	QL: 22. QT: 22.
**23**	Naringenin 7-*O*-rutinoside	98	BioCrick (BCN6300)	QL: 23. QT: 23.
**24**	Luteolin 3′-*O*-glucoside	95	PhytoLab (85840)	QL: 24. QT: 24.
**25**	Apigenin 7-*O*-rutinoside	98	Extrasynthese (1121 S)	QL: 25. QT: 25.
**26**	Naringenin 7-*O*-glucoside	95	Sigma (SMB00076)	QL: 26. QT: 26.
**27**	Luteolin 7-*O*-(6′′-acetyl)-glucoside	95	Lab collection/isolated from *D. palmatum* [27]	QL: 27. QT: 27.
**28**	Apigenin 7-*O*-glucoside	97	Sigma (44692)	QL: 28. QT: 45, 28.
**30**	Rosmarinic acid	99	Extrasynthese (4957 S)	QL: 30. QT: 30, 47.
**32**	Lithospermic acid B	98	BioCrick (BCC8249)	QL: 32. QT: 31, 32.
**33**	Lithospermic acid A	98	BioCrick (BCN5369)	QL: 33. QT: 33.
**36**	Eriodictyol	95	Sigma (74565)	QL: 36. QT: 36.
**37**	Luteolin	98	Sigma (L9283)	QL: 37. QT: 37.
**40**	Naringenin	95	Sigma (N5893)	QL: 40. QT: 40.
**41**	Apigenin	95	Sigma (10798)	QL: 41. QT: 41.
**42**	Apigenin 7-*O*-(4′′-malonyl-6′′-acetyl)-glucoside	95	Lab collection/isolated from *Matricaria chamomilla* [56]	QL: 42. QT: 42.
**43**	Acacetin 7-*O*-glucoside	95	ChemFaces (CFN92764)	QL: 43. QT: 29, 35, 43.
**44**	Apigenin 7-*O*-(6′′-*O*-acetyl)-glucoside	95	Lab collection/isolated from *D. palmatum* [27]	QL: 44. QT: 44.
**46**	Schizotenuin A	95	Lab collection/isolated from *Nepeta multifida* [39]	QL: 46. QT: 46.
**50**	Nepetamultin A	95	Lab collection/isolated from *N. multifida* [39]	QL: 50. QT: 48-50.
	Benzoic acid	99	Sigma (242381)	QT: 38, 39.

* Manufacturers list: BioCrick Co. Ltd. (Chengdu Tianfu, Sichuan, PRC); ChemFaces (Wuhan, Hubei, PRC); Extrasynthese (Lyon, France); PhytoLab GmbH & Co. KG (Vestenbergsgreuth, Germany); Sigma-Aldrich (St. Louis, MO, USA).

Table S2. Regression equations, correlation coefficients (r^2), standard deviation (S_{YX}), limits of detection (LOD), limits of quantification (LOQ) and linear ranges for 34 reference standards.

Compound	Ionization [a]	CE [b] (eV)	Regression equation [c]		r^2	S_{yx}	LOD/LOQ (µg/mL)	Linear range (µg/mL)
			a	$b \cdot 10^6$				
Caftaric acid	N	-20	1.4238	-0.0891	0.9901	$7.33 \cdot 10^{-2}$	0.17/0.52	0.6–100.0
Danshensu	N	-20	3.022	-0.769	0.9990	$1.02 \cdot 10^{-2}$	0.011/0.03	0.03–250.0
4-*O*-Caffeoylquinic acid	N	-15	0.9217	-0.0437	0.9982	$3.94 \cdot 10^{-2}$	0.14/0.43	0.5–100.0
4-Hydroxybenzoic acid 4-*O*-glucoside	N	-20	1.5379	-0.6220	0.9990	$0.99 \cdot 10^{-2}$	0.02/0.06	0.10–250.0
Eriodictyol 7-*O*-rutinoside	N	-20	4.1069	-0.5637	0.9989	$0.77 \cdot 10^{-2}$	0.006/0.02	0.02–400.0
Luteolin 7,4′-di-*O*-rutinoside	N	-20	2.0384	-0.3640	0.9975	$2.02 \cdot 10^{-2}$	0.03/0.10	0.10–350
5-*O*-Caffeoylquinic acid	N	-15	0.9406	-0.0497	0.9973	$5.18 \cdot 10^{-2}$	0.18/0.55	0.6–100.0
3-*O*-Caffeoylquinic acid	N	-15	0.9320	-0.0523	0.9991	$4.14 \cdot 10^{-2}$	0.15/0.44	0.5–100.0
1,3-Di-*O*-caffeoylquinic acid	N	-20	1.8535	0.0761	0.9989	$4.55 \cdot 10^{-2}$	0.08/0.25	0.3–100.0
Caffeic acid	N	-20	2.4493	-0.0938	0.9989	$1.85 \cdot 10^{-2}$	0.03/0.08	0.1–100.0
Luteolin 7-*O*-rutinoside-4′-*O*-glucoside	N	-20	7.833	-1.442	0.9984	$2.63 \cdot 10^{-2}$	0.011/0.03	0.04–500.0
Eriodictyol 7-*O*-glucoside	N	-20	4.9634	-0.5047	0.9972	$0.63 \cdot 10^{-2}$	0.004/0.01	0.01–400.0
Luteolin 7-*O*-rutinoside	N	-25	2.5078	-0.6342	0.9991	$0.96 \cdot 10^{-2}$	0.01/0.03	0.04–400.0
Luteolin 7-*O*-glucoside	N	-20	7.064	-1.533	0.9992	$1.92 \cdot 10^{-2}$	0.009/0.003	0.003–500.0
Luteolin 4′-*O*-glucoside	N	-20	1.4689	-0.3641	0.9990	$5.69 \cdot 10^{-2}$	0.12/0.38	0.40–400.0
Naringenin 7-*O*-rutinoside	N	-20	2.6340	-0.2411	0.9973	$2.74 \cdot 10^{-2}$	0.03/0.10	0.10–350.0
Luteolin 3′-*O*-glucoide	N	-20	1.1541	-0.4691	0.9987	$1.06 \cdot 10^{-2}$	0.03/0.10	0.10–350.0
Apigenin 7-*O*-rutinoside	N	-25	1.9634	-0.7458	0.9963	$2.59 \cdot 10^{-2}$	0.04/0.14	0.20–350.0
Naringenin 7-*O*-glucoside	N	-20	1.2716	-0.7389	0.9897	$9.14 \cdot 10^{-2}$	0.23/0.72	0.80–400.0
Luteolin 7-*O*-(6′′-acetyl)-glucoside	N	-25	7.804	-1.202	0.9944	$3.04 \cdot 10^{-2}$	0.012/0.04	0.04–250.0
Apigenin 7-*O*-glucoside	N	-20	5.802	-0.804	0.9990	$1.14 \cdot 10^{-2}$	0.007/0.02	0.02–500.0
Rosmarinic acid	N	-20	1.9610	-0.5271	0.9993	$0.94 \cdot 10^{-2}$	0.02/0.05	0.05–250.0
Lithospermic acid B	N	-25	1.706	-0.485	0.9963	$0.79 \cdot 10^{-2}$	0.015/0.05	0.05–250.0
Lithospermic acid A	N	-25	1.933	-0.562	0.9954	$0.83 \cdot 10^{-2}$	0.014/004	0.04–250.0
Eriodictyol	N	-20	3.6748	-0.7069	0.9987	$0.90 \cdot 10^{-2}$	0.008/0.02	0.02–400.0
Luteolin	N	-15	7.064	-1.533	0.9992	$1.92 \cdot 10^{-2}$	0.009/0.03	0.03–500.0
Naringenin	N	-20	1.1105	-0.3211	0.9937	$4.18 \cdot 10^{-2}$	0.12/0.38	0.40–400.0
Apigenin	N	-25	5.206	-1.407	0.9992	$1.52 \cdot 10^{-2}$	0.010/0.03	0.03–500.0
Apigenin 7-*O*-(4′′-malonyl-6′′-acetyl)-glucoside	N	-20	1.9871	-0.6871	0.9984	$5.63 \cdot 10^{-2}$	0.09/0.28	0.40–400.0
Acacetin 7-*O*-glucoside	N	-25	1.1492	-0.6010	0.9980	$4.68 \cdot 10^{-2}$	0.14/0.41	0.50–400.0
Apigenin 7-*O*-(6′′-*O*-acetyl)-glucoside	N	-30	5.534	-0.705	0.9953	$2.01 \cdot 10^{-2}$	0.012/0.04	0.04–250.0
Schizotenuin A	N	-25	2.733	-0.637	0.9991	$0.52 \cdot 10^{-2}$	0.006/0.02	0.02–250.0
Nepetamultin A	N	-25	2,536	-0.473	0.9962	$0.64 \cdot 10^{-2}$	0.008/0.03	0.03–250.0
Benzoic acid	N	-10	1.5379	-0.6220	0.9990	$0.99 \cdot 10^{-2}$	0.02/0.06	0.10–250.0

[a] Ionization mode: N – negative. [b] CE – collision energy. [c] Regression equation: $y = a \cdot x + b$
